# Ultra-High Contact Electrified Current Generation and Chemical Sensing at IL-Based Immiscible Liquid–Liquid Interface

**DOI:** 10.3390/mi17060688

**Published:** 2026-06-02

**Authors:** Yunfei Deng, Junyan Zhang, Hongmian Qi, Shaobin Wen, Chengfa Wang, Zhe Yu, Mengqi Li

**Affiliations:** 1 Marine Engineering College, Dalian Maritime University, Dalian 116026, Chinawangcf08@dlmu.edu.cn (C.W.);; 2 Liaoning Ocean and Fisheries Science Research Institute, Dalian 116023, China

**Keywords:** contact electrification, charge transfer, ionic liquid, liquid–liquid interface, chemical sensing

## Abstract

Though the charge transfer efficiency of a liquid–liquid (L-L) nanogenerator is much higher than that of the solid–liquid and solid–solid counterparts, there is still much room for improving the level of interfacial charge transfer. In this study, a power generation system was designed using an ionic liquid (IL)-based immiscible L-L interface via the contact/separation mode. The maximum output electric current reaches as high as about 8.12 μA by contacting a saturated NaCl droplet with an immiscible IL droplet. The magnitude of the generated electric current signals increases with an increase in the ionic concentration of the NaCl solution, the droplet contact area, and the liquid volume of IL. Moreover, the magnitude of the signals varies slightly when the pH value is under 12 and increases sharply in strong alkaline conditions. A maximum instantaneous power output of about 82 nW was obtained with a 100 kΩ resistor in series. The IL-based immiscible L-L interface configuration has been proven to be capable of sensing metal ions at an ultra-low concentration via the contact/separation mode.

## 1. Introduction

A nanogenerator (NG) is a device that converts ambient mechanical energy into electrical energy based on contact electrification and electrostatic induction. NGs show great potential for self-powered sensing (eliminating the need for external batteries) [1,2,3], and signal transmission [4] and communication [5], particularly in remote, implantable, or wearable applications where sustainable and maintenance-free operation is critical. The mechanism of contact electrification (CE) refers to the charge transfer between two different materials [6,7], where net positive charges accumulate on one material and net negative charges on the other [8]. Based on the working principle of the interfacial electrical phenomena, the traditional methods for electricity generation are designed based on a solid–solid interface and can be generally divided into three types: piezoelectric (PENG), triboelectric (TENG), and piezoionic.

The piezoelectric method works on the piezoelectric effect, where a voltage difference across the piezoelectric material is generated as it is stressed [9]. Generating electricity based on the triboelectric effect has also been proposed, which mainly depends on different friction materials as they have different abilities to gain or lose electrons during the friction process [10]. Distinct from the electricity generated by the piezoelectric effect or triboelectric effect using electrons as a charge carrier, electricity can also be generated by the so-called piezoionic effect, which makes use of ionic transport to produce an electrical output upon an applied pressure [11,12].

As a method of harvesting ambient mechanical energy from water movement (such as waves, raindrops, flows, and evaporation), electricity generation via a solid–liquid interface has recently attracted considerable attention [13,14,15]. Various approaches have been investigated to generate electricity using the charge transfer at the solid–liquid interface; the main ones include liquid motion [16], waving [17], twisting [18], and water evaporation [19]. The mechanism of electricity generation can be attributed to capacitive discharge based on the establishment and disappearance of the electric double layer (EDL) capacitance [19]. Take the electricity generation via liquid motion on solid surface as an example: the drawing potential is generated by the propagation of the solid–liquid boundary [20], where mobile charges in the EDL form at the front forwarding boundary and vanish at the rear releasing boundary [21]. In addition, the liquid flow-induced voltage and current generation are found in various nonstructural materials such as copper oxide [22], graphene oxide-polypyrrole [23], self-assembled monolayer [24], reduced graphene oxide [25], carbon nanotube [26], and graphene [27].

Though designs of solid–solid interfaces and solid–liquid interfaces have been largely developed in recent years, the non-flexibility of the solid section hinders the total contact between the two phases. The effective contact area of a liquid–liquid (L-L) interface offers a promising solution to this issue. In 2019, Nie et al. [28] proposed the first L-L TENG. A falling droplet passing through a pre-charged membrane can generate a peak electric current of about 60 nA. In the same year, Song et al. [29] reported electricity generation via the coalescence of a water droplet with an air–liquid interface. Nevertheless, the peak value of electric current is only about 350 nA. Though the miscible L-L interface can be used as L-L TENG, the output efficiencies of these electric current generation devices are relatively low. To increase the charge transferring efficiency, different types of immiscible L-L interfaces were proposed. For example, by dropping deionized (DI) water into transformer oil, an electric current of 40 pA was achieved [30]. More recently, the PEG droplet and DEX droplet were employed as the two immiscible aqueous phases [31]. Due to their immiscibility, the two droplets can be brought into contact and separate effectively, obtaining a peak electric current of 720 nA. Nevertheless, the magnitude of the electric current generated by these immiscible L-L interfaces is still limited.

As reviewed above, there is still much room for improving the electric output via the immiscible L-L interface. In this work, an IL-based immiscible L-L interface was proposed to generate an ultra-high output electric current via the contact/separation mode. An experimental system was developed that included two hollow electrodes for loading IL droplet and electrolyte droplets, and an electrometer with home-made LabVIEW software (LabVIEW 18.0 development system). The effects of the ionic concentration of the electrolyte solution, droplet contact area, pH value, liquid volume of IL, and load resistor on the signal magnitudes were systematically investigated. The sensing of different metal ions at an ultralow concentration via the contact/separation mode by the IL-based immiscible L-L interface configuration was also demonstrated. It should be emphasized that the electric current generated by the IL-based immiscible L-L interface (8.12 µA) is, to the best of our knowledge, the largest ever reported for any liquid–liquid triboelectric nanogenerator, surpassing the previous record of 720 nA by the PEG-DEX interface. This significant improvement is attributed to the ultra-high ion density and stable immiscibility of the IL with water.

## 2. Experimental System and Working Principles

**Figure 1:**
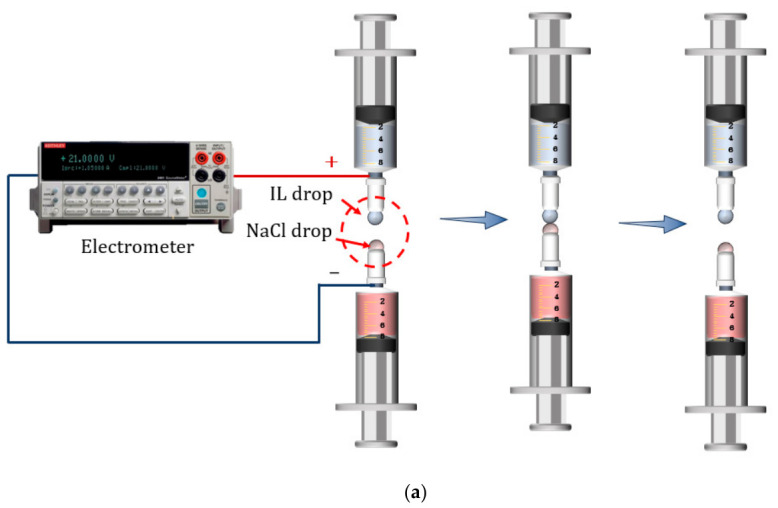

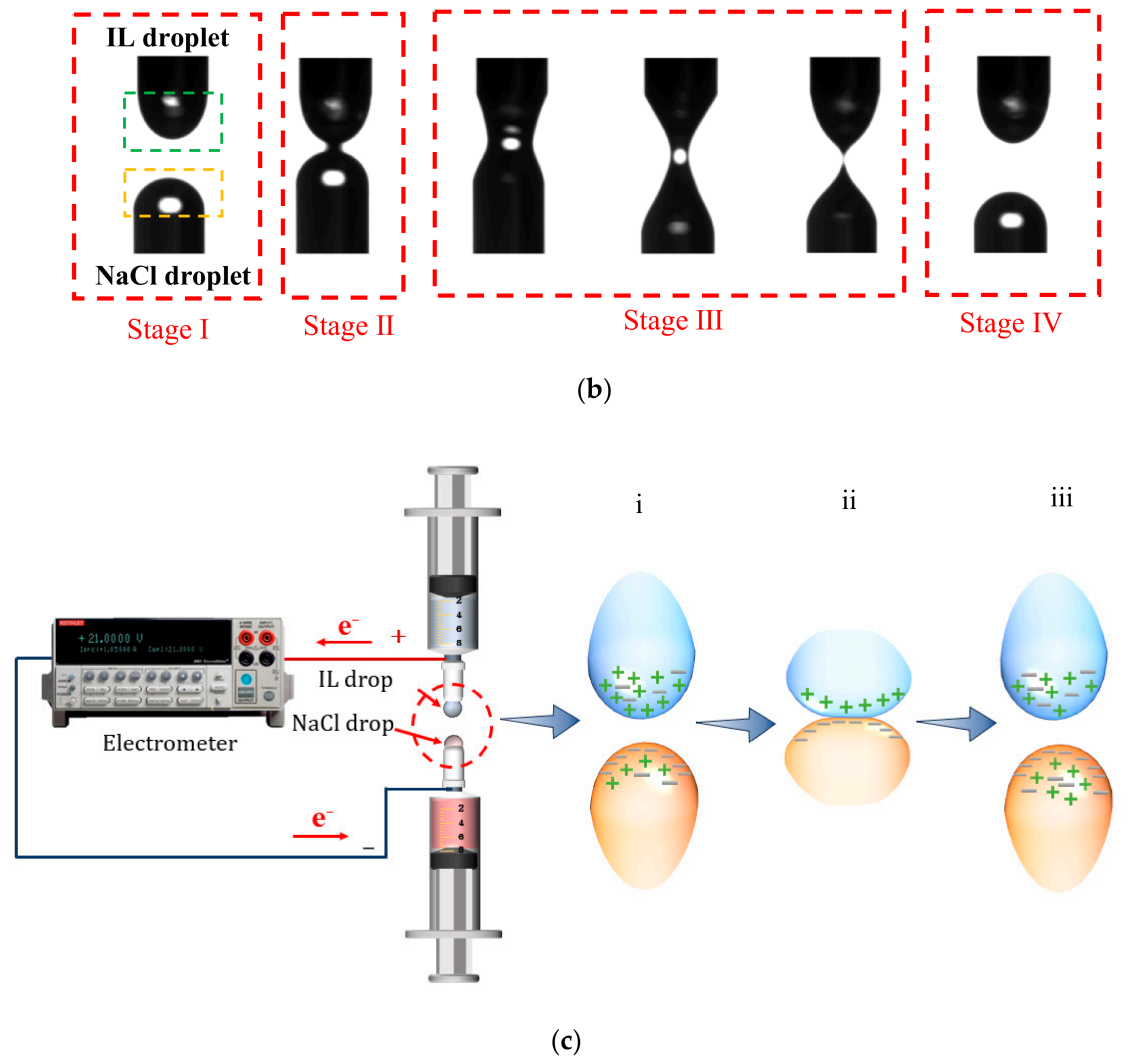
(**a**) Schematic diagram of the ultra-high contact electrified current generation system at the IL-based immiscible L-L interface; (**b**) photograph of the contact and separation process of the two droplets (Stage I: before contact, Stage II: in contact, Stage III: toward separation, Stage IV: separated); (**c**) schematic diagram of the working mechanism of the IL-based immiscible L-L interface current generation.

Figure 1a shows a schematic diagram of the ultra-high contact electrified current generation via the IL-based immiscible L-L interface. The system mainly includes two syringes with hollow electrodes (flat-headed stainless-steel needles), an electrometer and a computer with home-made LabVIEW software to record the data measured with the electrometer. Of the two syringes with hollow electrodes, one is filled with IL and the other with electrolyte solution. Specifically, the hollow electrode filled with IL is connected to the positive terminal of the electrometer, and that filled with electrolyte solution is connected to the negative terminal of the electrometer. The hollow electrodes and the electrometer are connected by copper electric wires. As the viscosity of the IL is much higher than that of the electrolyte solution, the IL is loaded on the upper hollow electrode, and the electrolyte solution is loaded on the lower hollow electrode. Before the process of electric current generation, the extruded volumes of the two liquids are carefully controlled to form semicircular droplets. Then, the hollow electrode filled with electrolyte solution is moved up and down periodically by a stepping motor to achieve contact and separation with the IL droplet.

To verify the feasibility of continuous power generation by the immiscible L-L interface, the immiscibility of the IL droplet and electrolyte droplet during the contact and separation process should be confirmed. Photographs of the two droplets during the contact and separation process are shown in Figure 1b. It can be seen that before the contact of the two droplets (Stage I), the pendant IL droplet and the upward electrolyte droplet are semicircular, as described above. Once the electrolyte droplet is moved up to contact the IL droplet, it can be clearly seen that there is a liquid bridge between the L-L interface (Stage II). When the electrolyte droplet is moved downward to separate from the IL droplet (Stage III), the interface of the two droplets is deformed at the same time. Although the viscosity of the IL is relatively high and has aims to carry certain volumes of the NaCl droplet, it can be clearly seen in Stage IV that the morphology and volume of the two droplets basically did not change during the contact and separation. The result indicates that the two droplets did not dissolve with each other during the contact and separation process.

The working mechanism of the electric current generation system based on the immiscible L-L interface is illustrated in Figure 1c. It should be noted that for the electrolyte droplet, the air–electrolyte surface is negatively charged due to the preferential adsorption of hydroxide ions (OH^−^) or the orientation of water dipoles at the interface [32,33]. Nevertheless, as the IL droplet is fully filled with cations and anions, the concentration of either cations or anions is much larger than that of the electrolyte droplets because no water molecules are included. In other words, the electric double layer (EDL) formed at the IL–fluid interface is much denser than conventional electrolyte–fluid interfaces [34]. Therefore, the electric double layer (EDL) at the IL–fluid interface is much denser, leading to a larger surface charge density compared to the air–electrolyte surface.

When the IL droplet and the electrolyte droplet are separated, as shown in Figure 1c-i, the two droplets are electrically neutral and there is no electron migration in the external circuit. Therefore, no electric current or voltage signals can be detected. When the electrolyte droplet is brought into contact with the IL droplet (Figure 1c-ii), surface charges on the interfaces of the two droplets are redistributed as the negatively charged electrolyte droplet will attract the cations from the IL droplet to the surface and repel the anions from the surface. Therefore, an electric potential difference (∆*V*) at the L-L interface is formed and the charges (*Q*) migrate from the electrolyte droplet to the IL droplet, which can be expressed as Q=σ·S, where *σ* is the surface charge density determined by the surface potential and the concentration of mobile ions, and *S* is the contact surface area.

As the IL droplet and the electrolyte droplet are immiscible, the interface of the two droplets can be regarded as infinitely thin. The ions with opposite signs at the interface allow an effective capacitance change upon the contact. Therefore, the immiscible L-L interface in our system can be approximately equalized to a capacitor [34,35]:

(1) $$C=\frac{\varepsilon_{r}\varepsilon_{0}S}{d}$$

where $\varepsilon_{r}$ is the effective relative permittivity of the medium between the two droplets (a weighted average of the permittivities of the IL and the electrolyte at the interface), and d is the distance between the two droplets. According to the definition of the capacitance (*C*), the transferred charges (*Q*) at the L-L interface can also be expressed as

(2) Q=C·ΔV

Since the hollow electrode filled with IL is connected to the positive terminal of the electrometer, and that filled with electrolyte solution is connected to the negative terminal of the electrometer, the electric current (*I*) and electric potential difference (∆*V*) detected by the electrometer are both positive, accordingly generating a positive electric current:

(3) $$I=\frac{dQ}{dt}=\frac{d(C\cdot \Delta V)}{dt}\approx \Delta V\frac{dC}{dt}$$

Combining the above equations, the generated electric current can be expressed as

(4) $$I=\Delta V\frac{dC}{dt}=\frac{\Delta V\varepsilon_{r}\varepsilon_{0}S}{d\Delta t}$$

It can be found that the magnitude of the electric current is determined by the electric potential difference (∆*V*) at the L-L interface, contact surface area (*S*), the distance between the two droplets, etc. When the IL droplet is detached from the electrolyte droplet (Figure 1c-iii), the charges are restored to the initial stage, as is the case before contact with each other.

## 3. Experimental Setup

### 3.1. Materials

In this study, 1-hexyl-3-methylimidazolium hexafluorophosphate (HMIMPF6, Yi En chemical technology Co., Ltd., Shanghai, China) was selected to form the IL droplet, which is insoluble in water. Electrolyte solution was prepared by diluting a certain amount of sodium chloride (NaCl, 99.5% purity, Tianjin Zhiyuan, Tianjin, China) in purified water. In addition, electrolyte solutions with different pH values were configured with oxalic acid (C_2_H_2_O_4_, 99.99% purity, aladdin, Shanghai, China) and sodium hydroxide (NaOH, purity ≥99%, Thermo Fisher Scientific, Shanghai, China) to investigate the effect of pH on electrified current generation. FeCl_3_ solution, PbCl_2_ solution, and CuCl_2_ solution were prepared by adding certain amounts of iron chloride (FeCl_3_, 99.99% purity, aladdin, Shanghai, China), lead chloride (PbCl_2_, 99.99% purity, aladdin, Shanghai, China), or copper chloride (CuCl_2_, 99.99% purity, aladdin, Shanghai, China) to purified water. The syringes and the flat-headed stainless-steel needles were purchased from the local pharmacy. The two needles are connected in series with an electrometer (6517B, Keithley, Beaverton, OR, USA). A home-made LabVIEW program was used to record the real-time electric current signals measured with the electrometer.

### 3.2. Experimental Procedures

In this study, the effects of the ionic concentration, contact area of the two droplets, droplet volume, electrode area, and pH value of the electrolyte solution were investigated. To start the experiments, the upper hollow electrode was filled with IL to form a pendant IL droplet, and the lower hollow electrode was filled with NaCl solution to form an electrolyte droplet. We adjusted the scale of the syringe to ensure that the volume of the two droplets was approximately equal. We moved the lower hollow electrode up and down with the stepping motor to achieve contact and separation with the upper IL droplet. The electric current and voltage signals were collected by the electrometer and recorded with the home-made LabVIEW software. The above processes were repeated three times, and the generated electric signals were obtained by averaging the three measurements. All experiments were conducted at room temperature (23 ± 1 °C).

## 4. Results and Discussion

### 4.1. Typical Electric Signals

**Figure 2:**
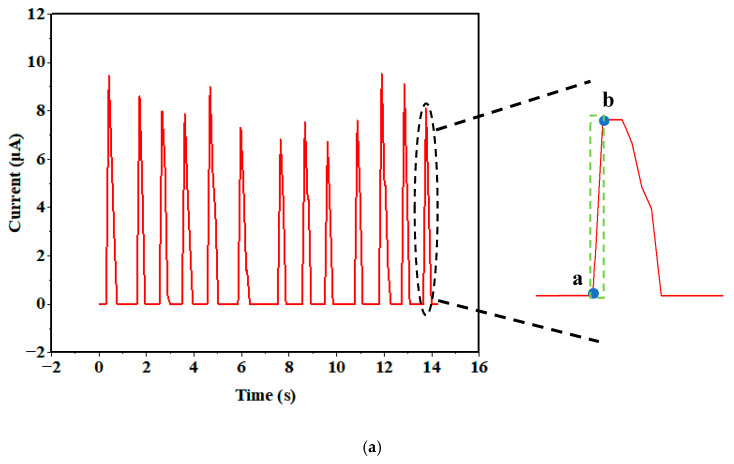

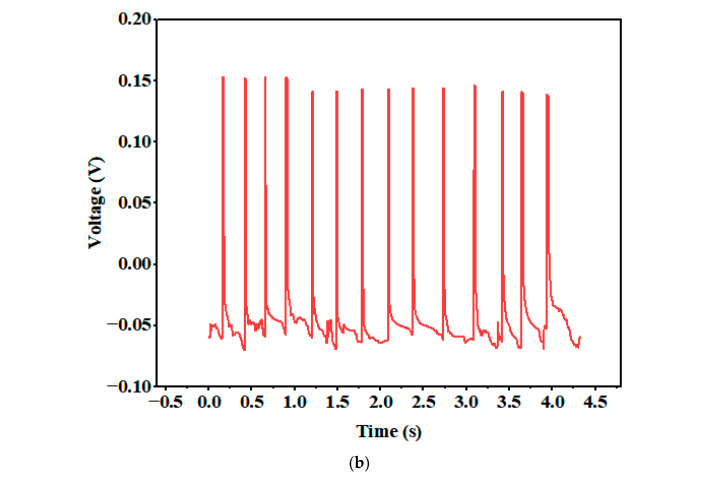
Typical electric current signals (without external resistor) (**a**) and voltage signals (**b**) generated by the contact of an IL droplet and a saturated NaCl droplet.

As described in Section 2, the electric current signals should be positive in our system. To verify this prediction and test the performance of the IL-based ultra-high contact electric current generation system, a saturated NaCl droplet was placed in contact with a pendant IL droplet. The sectional area of the flat-headed needles (G10, 9.61 mm^2^) for the two droplets remained unchanged, and volumes of the droplets were controlled to be almost the same during the experiment. The experimental results of the generated electric current are shown in Figure 2a. The electric current signals generated by contacting the IL droplet with the saturated NaCl droplet are positive, which means that the electric current flows from the IL droplet to the NaCl droplet. Moreover, the average magnitude of the generated current can reach as high as 8.12 μA. As compared with the recently reported electric current generation by the L-L interfaces in Table 1, the largest magnitude of the generated electric current was about 720 nA by the PEG-DEX interface [31], the electric current generated by our immiscible L-L interface in this study is over 10 times larger than the electric current generated by the PEG-DEX interface.

We can also observe from the amplified signal in Figure 2a that as the two droplets begin to contact (point a), the electric current also begins to increase, reaching a peak rapidly at point b. The response time of the electric current generation is about 38 ms as calculated from point a to point b. Then, the two droplets begin to separate, and the electric current begins to decrease sharply to 0. It should be noted that the pulse of the electric current is generated at the moment the two droplets make contact. After the contact, the electric current restores to the baseline even if the two droplets are still in contact. Similarly, when two droplets begin to make contact, the voltage also begins to increase. It can be seen from Figure 2b that the average measured voltage is about 0.23 V. As the contact area increases until the two droplets are in complete contact, the voltage reaches a peak value. After that, the two droplets begin to separate, and the voltage also begins to decrease to 0.

It can be seen that only positive current signals are detected in our system, with no observable reverse pulse upon separation. This is because our system operates in a “contact-only” detection mode—the signal is recorded at the moment of contact when dC/dt is positive and large. Upon separation, dC/dt is negative but small in magnitude due to the slow separation speed and the stable EDL at the IL-fluid interface, resulting in a gradual return to baseline without a distinct negative peak. This behavior is consistent with the model in Equation (4), where the current is proportional to dC/dt.

It should be noted that when directly shorting the red and black clips of our Keithley 6517B under identical measurement settings, the measured baseline offset was approximately 0.2–0.3 µA, revealing a small offset artifact typical of electrometers when measuring low-impedance sources. As discussed above, the initial measurements of the IL-based ultra-high contact electric current generation system using an electrometer showed an apparent current of 8.12 µA, which is more than 25 times larger than this artifact. Therefore, the corrected peak current should be 0.2–0.3 µA smaller than the data shown on the display of the Keithley 6517B.

Based on the measured peak current (7.8 µA) and pulse width (38 ms), the total transferred charge is approximately 3.0 × 10^−7^ C. With a contact area of 9.61 mm^2^, the calculated surface charge density is 3.1 × 10^−2^ C/m^2^. This value is comparable to (and slightly higher than) the 1.81 × 10^−2^ C/m^2^ reported for the PEG-DEX liquid–liquid interface [31], which is expected given the much higher ion density of the ionic liquid used in our work.

To further verify that the measured signal is not a constant instrumentation artifact, we performed two additional control experiments. First, we exchanged the connections of the electrometer (positive terminal to the NaCl droplet, negative terminal to the IL droplet); under this configuration, the polarity of the current signal reversed from positive to negative, confirming that the signal is genuinely related to the contact between the two droplets. Second, we replaced the IL droplet with a second saturated NaCl droplet (same material on both sides); under this configuration, no significant current signal was generated upon contact, further confirming that the signal arises from the surface charge difference between the two dissimilar liquids.

### 4.2. Effect of Ionic Concentration on Electric Signals

**Figure 3:**
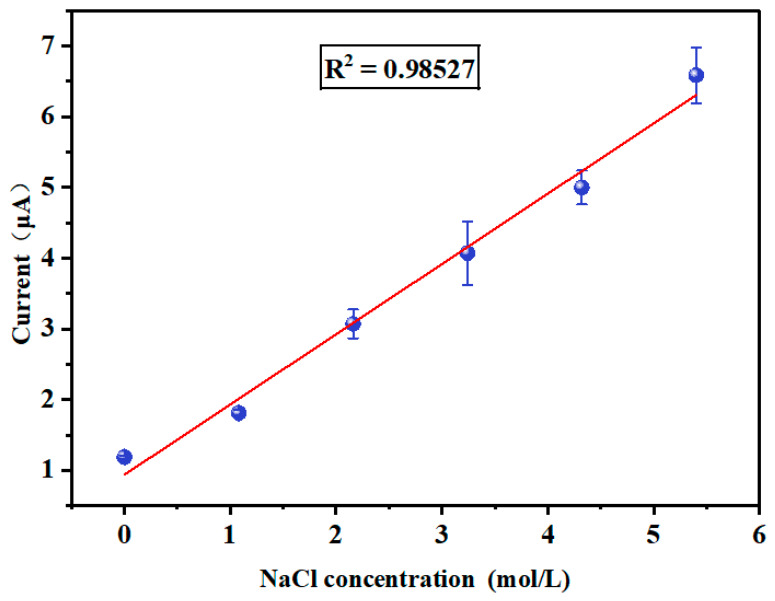
Effect of ionic concentration on the electric current signals (without external resistor).

As discussed in Section 2, the surface charge density of the NaCl droplet will affect the magnitude of the generated electric current signals. To verify this prediction, different concentrations (5.43 mol/L, 4.32 mol/L, 3.24 mol/L, 2.16 mol/L, and 1.08 mol/L) of NaCl solutions were prepared by diluting the saturated NaCl solution with different amounts of purified water. Specifically, purified water was also employed to prepare the solution of zero ionic concentration. It should be noted that in this section, the IL is kept unchanged as it is made up of total cations and anions. The experimental results are shown in Figure 3. It can be seen that with the increase in the ionic concentration of NaCl solution, the electric current signal generated by the contact and separation of the NaCl droplet and the IL droplet shows a linearly increasing relationship, and the direction of the electric current is positive. For example, the electric current was about 1.78 μA when the ionic concentration of the NaCl solution was 1.08 mol/L. When the ionic concentration of the NaCl solution is increased to 5.4 mol/L, the electric current reaches a peak of about 6.59 μA. The surface charge density of the electrolyte droplet increases with the increase in the ionic concentration of the NaCl solution. According to the equation Q=σ·S, the transferred charges increase as well, resulting in an increase in the electric current when the two droplets contact each other.

### 4.3. Effect of L-L Contact Area on Electric Signals

**Figure 4:**
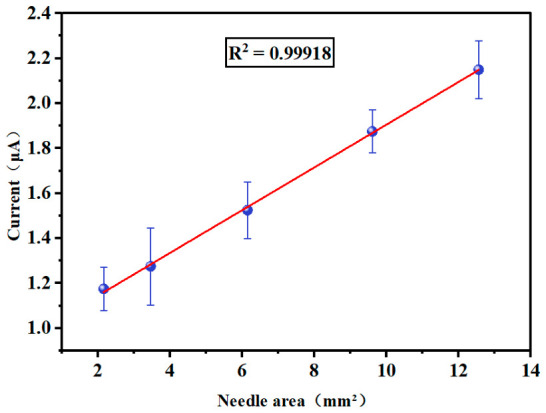
Dependence of electric current signals on needle area (without external resistor).

As predicted in Equation (3), the generated electric current is dependent on the contact area of the IL droplet and the NaCl droplet. In the experiments, the droplet surface area increases with the increase in the flat-headed needles. Therefore, the influence of droplet contact area on the power generation was studied by adjusting the diameter of the needles for loading the IL droplet and the NaCl droplet to verify this prediction. The ionic concentration of the NaCl solution is 1.08 mol/L. Flat-headed needles with different sectional areas (2.16 mm^2^, 3.46 mm^2^, 6.15 mm^2^, 9.61 mm^2^, and 12.56 mm^2^) are used to vary the contact area between the IL droplet and the NaCl droplet. It should be noted that the contact area of the two droplets is proportional to the sectional area of the needles. The experimental results are shown in Figure 4. It can be seen that as the needle sectional area increases from 2.16 mm^2^ to 12.56 mm^2^, the magnitude of the electric current linearly increases from 1.18 μA to 2.15 μA. This experimental result agrees well with our theoretical analysis. Therefore, it can be predicted that the electric current generation performance of our IL-based immiscible L-L interface can be significantly improved by optimizing the surface area of the electrode design.

### 4.4. Effect of Liquid Volume on Electric Signals

**Figure 5:**
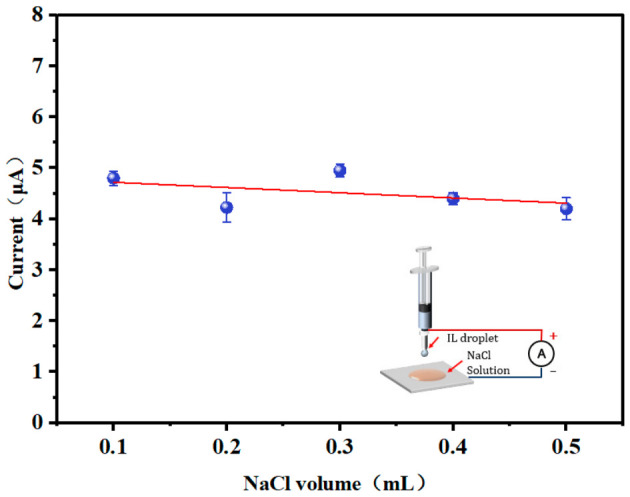
Effects of NaCl volume on the generated signals (without external resistor).

To verify the effect of surface charges of NaCl solution and IL on the performance of the electric current generation, different volumes (0.1 mL to 0.5 mL) of saturated NaCl solution and IL were individually dropped on a copper plate electrode to contact with the pendant droplet (IL or NaCl). It should be noted that the spread area of either NaCl or IL on the copper plate electrode is smaller than that of the copper plate electrode. The pendant droplets are formed using a G10 flat-headed needle. It can be seen from Figure 5 that as the volume of the saturated NaCl solution on the copper plate electrode increases, the generated electric current is almost unchanged. For example, when the volume of the saturated NaCl solution is 0.1 mL and 0.5 mL, the current is 4.95 μA and 4.2 μA, respectively. It can be concluded that the electric current generation of IL-based immiscible L-L interface is independent of the volume of the saturated NaCl solution.

This phenomenon can be understood as follows: A larger volume of the NaCl solution on the copper plate electrode means a larger surface area of the NaCl solution. The amount of the total surface charges should increase as well. Nevertheless, as the IL droplet and the NaCl solution on the copper plate electrode are immiscible, the total surface charges transferred should be dependent on the capacitance model, where the effective contact surface area determines the generated electric current. It is not difficult to understand why the generated electric current is almost unchanged with the increase in the NaCl solution volume.

### 4.5. Effect of pH Values on Electric Signals

**Figure 6:**
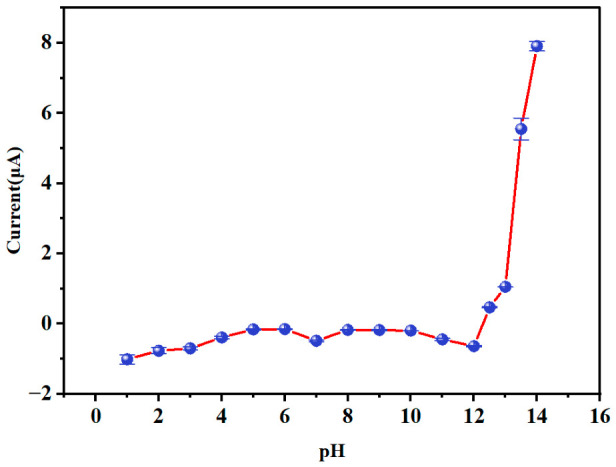
Effect of pH values on the generated electric current signals (without external resistor).

Generally, the interfacial electric potential is related to the pH value of the solution. In this section, NaCl droplets with different pH values were prepared to contact with the pendant IL droplet to explore the effect of pH values on the generated electric signals. NaCl droplets with different pH values were prepared in pure water by adding different concentrations of oxalic acid and sodium hydroxide. The experimental results are shown in Figure 6. It can be seen that in acidic solutions and weakly alkaline solutions, the current signals are negative, and the amplitude is almost equivalent to the current signal generated by the contact of the pure water droplet with the IL droplet. With an increase in the concentration of oxalic acid (decrease in pH values), the absolute value of the generated current signals increases in magnitude. Moreover, under weak alkaline conditions, the current signal generated decreases slightly in value as the pH increases to 12. It can be speculated that the presence of hydrogen ions and hydroxyl ions will affect the selective distribution of positive and negative ions in the NaCl phase near the L-L interface. When the pH value is in the strong alkaline range (pH = 13 and 14), the power generation effect increases significantly. Specifically, the magnitude of the generated current signal is about positive 8 μA at pH = 14. To further verify the sharp increase between pH 12 and pH 14, additional measurements were performed at pH 12.5 and 13.5. The results confirm a continuous and sharp increase in current under strongly alkaline conditions. This is attributed to the specific adsorption of OH^−^ ions, which increases the negative surface charge density of the electrolyte droplet, thereby enhancing the interfacial potential difference.

### 4.6. Effect of Load Change on Electric Signals

**Figure 7:**
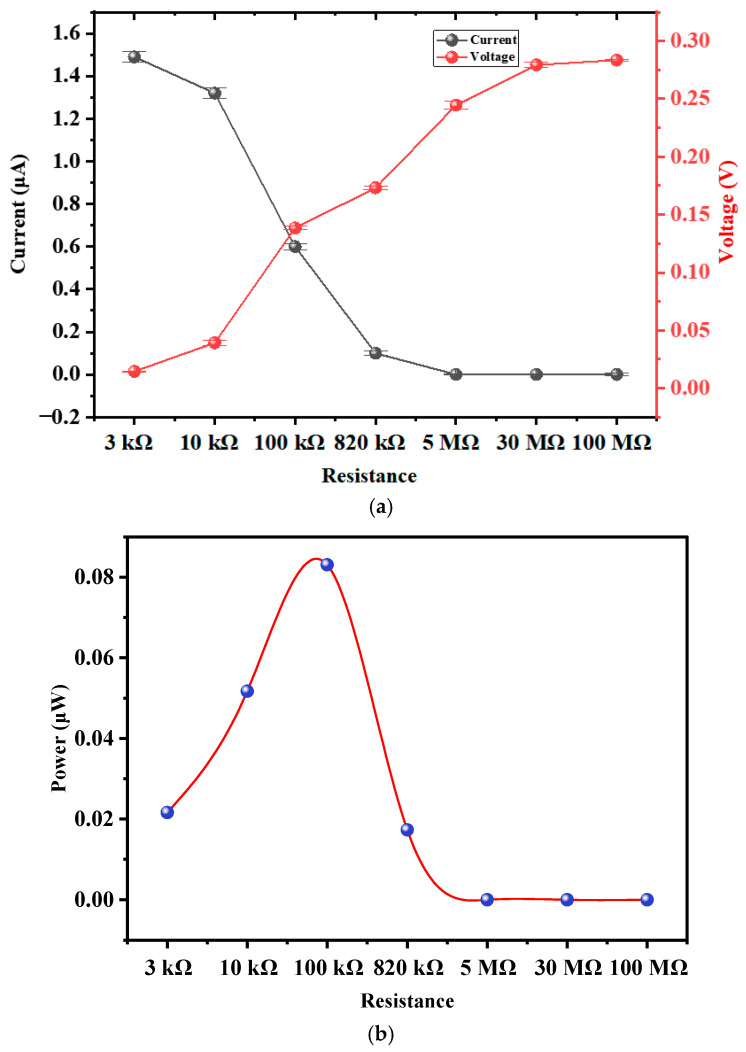
(**a**) The variation in current and voltage signals of the IL-based immiscible L-L interface with load resistance; (**b**) the relationship between the IL-based immiscible L-L interface power generation and the load resistance.

To characterize the electric output performance of the IL-based L-L interface electric current generation, the effect of load change on the electric output should be systematically investigated. Figure 7a shows the relationship of the generated electric current and voltage with the load resistor. It can be seen that as the resistance in series in the circuit increases, the voltage across the resistor shows an increasing trend. By contrast, the current in the circuit shows a downward trend. This phenomenon follows the basic principle of Ohm’s law. As the load resistance increases, the current in the circuit decreases due to the internal impedance of the generator. The measured voltage across the resistor initially increases with R and eventually saturates at the open-circuit voltage (≈0.28 V) when the current approaches zero. When the resistance exceeds 5 MΩ, the voltage across the resistor reaches a plateau value (between 0.24 V and 0.28 V), indicating that the maximum voltage generated by the two droplets has been reached. By contrast, the current in the circuit decreases to nearly zero when the resistance reaches 5 MΩ. The formula P = UI is used to obtain the instantaneous power curve. As shown in Figure 7b, the maximum instantaneous power can reach as high as about 82 nW, where the resistor in series is 100 kΩ, and the voltage and current can reach 0.13 V and 0.6 μA, respectively.

### 4.7. Charging of Commercial Capacitors

**Figure 8:**
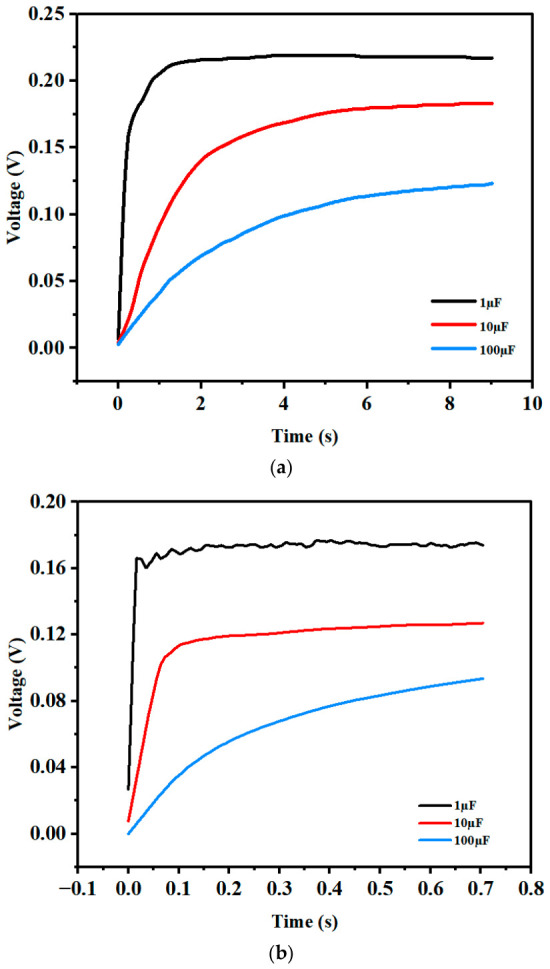
The charging curves of commercial capacitors (1 μF, 10 μF, and 100 μF) in series with a 1 MΩ resistor (**a**) or no resistor (**b**) in the circuit.

To verify the feasible application of IL-based immiscible L-L interface power generation, the system was used to charge commercial capacitors (1 μF, 10 μF, and 100 μF) with a 1 MΩ resistor or no resistor in series. As shown in Figure 8a, when the 1 MΩ resistor is connected in series in the circuit, commercial capacitors of 1 μF, 10 μF, and 100 μF can be charged to 0.22 V, 0.18 V, and 0.13 V, respectively. A 1 MΩ resistor was used in series to limit the charging current and to demonstrate the device’s ability to charge capacitors under a controlled resistive load. This allows for comparison of charging behavior under different load conditions. Without the resistor, charging is much faster (Figure 8b) but less controlled. For the case with no resistor in the circuit, the commercial capacitors of 1 μF, 10 μF, and 100 μF could be charged to 0.18 V, 0.13 V, and 0.11 V, respectively (Figure 8b). It should be noted that the charging process with no resistor is much faster than that of a 1 MΩ resistor in series. For example, for a 10 μF capacitor, the circuit with no resistor connected takes 0.1 s to fully charge, while the circuit with a 1 MΩ resistor in series takes 6 min to fully charge.

## 5. Chemical Sensing of Metal Ions

**Figure 9:**
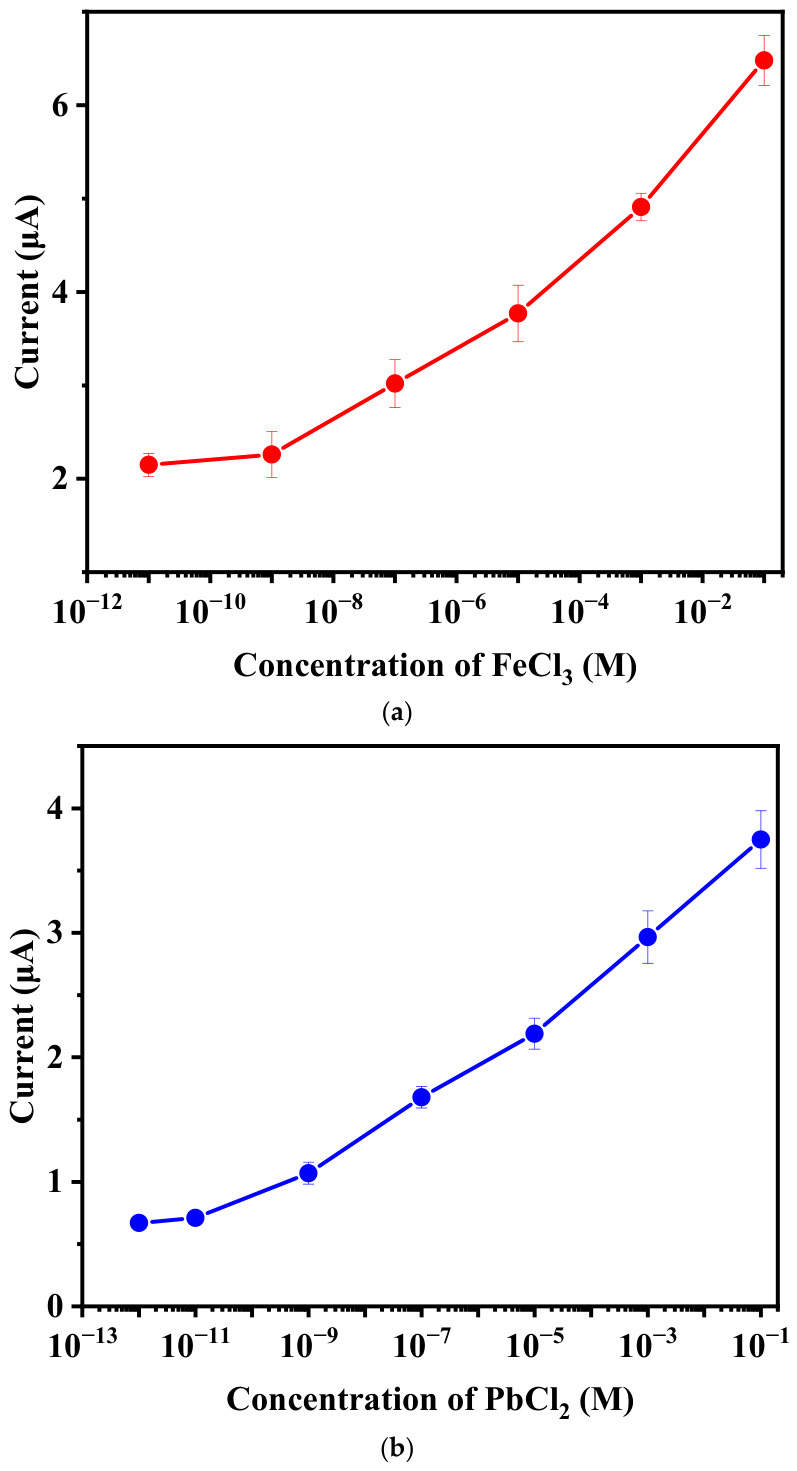

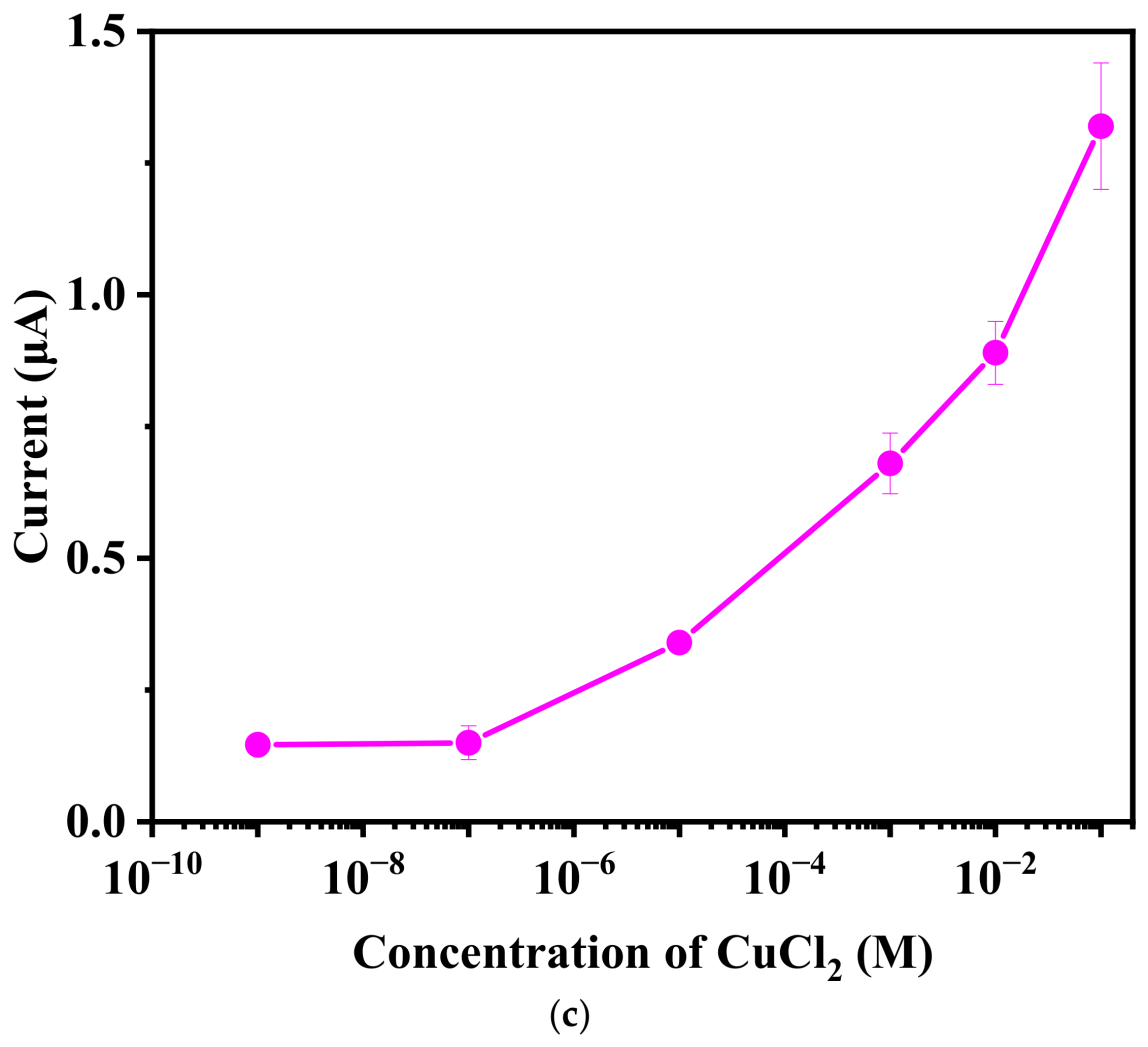
Chemical sensing of FeCl_3_ (**a**), PbCl_2_ (**b**), and CuCl_2_ (**c**) by IL-based immiscible L-L interface.

As discussed in Section 2, Equation (4) can be applied to various surface charge sensing fields by the generated electric signals with the immiscible L-L interface. For example, electrolyte droplets with different surface charge densities can be distinguished by contacting with the IL-based droplet. As previously discussed, the measured electric current signal is dependent on the interfacial electric potential difference between the two contacting droplets. In this section, metal ions with different concentrations were taken as an example to demonstrate the application of the IL-based immiscible L-L interface. Specifically, droplets with Fe^3+^, Pb^2+^, and Cu^2+^ were individually prepared to contact the IL droplet. The experimental results in Figure 9 show the dependence of the measured electric current signals on the concentration of the metal ions. It can be clearly seen that the generated electric current signals increase with an increase in the metal ion concentration. For example, the generated electric current signals increase from about 2.15 μA to 6.48 μA when the Fe^3+^ concentration increases from 10^−11^ M to 10^−1^ M. In addition, using our sensing method, the limit of detection (LOD) of the Fe^3+^, Pb^2+^, and Cu^2+^ was 10^−11^ M, 10^−12^ M, and 10^−9^ M, respectively. Compared with other detection methods [36,37], our method is also at the same level in terms of LOD for metal ion detection.

It should be noted that, based solely on the measured current magnitude, one cannot uniquely distinguish either the ion species or its concentration if the analyte is unknown. However, for a known target ion (e.g., Fe^3+^ in a calibrated system), the current signal can be used to determine its concentration. The LOD values reported (10^−11^ M for Fe^3+^, 10^−12^ M for Pb^2+^, and 10^−10^ M for Cu^2+^) are valid for the respective single-ion solutions under controlled conditions. For selective sensing in mixtures, additional strategies such as ion-selective membranes would be required.

## 6. Conclusions

In this study, a contact/separation power generation system was designed using an IL-based immiscible L-L interface. The magnitude of the generated electric current signals increases with an increase in the ionic concentration of the NaCl solution, the droplet contact area, and the liquid volume of IL. Moreover, the magnitude of the signals varies slightly when the pH value is under 12 and increases sharply in strong alkaline conditions. The maximum electric current can reach an ultra-high value of about 8.12 μA with a saturated NaCl droplet. A maximum instantaneous power output of about 82 nW was obtained with a 100 kΩ resistor in series. The demonstration of sensing different metal ions was achieved with the IL-based immiscible L-L interface electric generation system via contact/separation mode.

## Figures and Tables

**Table 1:** Output performance comparison of the liquid–liquid electrified interfaces.

Materials	Contact Interface	Voltage	Current	Power	Refs.
Liquid droplet–liquid membrane	Liquid–liquid	4 V	60 nA	137.4 nW	[28]
Ferrofluid–lubricant oil	Liquid–liquid	1.3 V	1 nA	—	[34]
Transformer oil–falling droplets	Liquid–liquid	—	40 pA	—	[30]
PEG-DEX	Liquid–liquid	0.4 V	720 nA	100 nW	[31]
NaCl droplet–NaCl droplet	Liquid–liquid	—	350.72 nA	—	[29]
This work	Liquid–liquid	0.23 V	8.12 μA	82 nW	

## Data Availability

The original contributions presented in this study are included in the article. Further inquiries can be directed to the corresponding author.

## References

[1] Chang Y., Wang L., Li R., Zhang Z., Wang Q., Yang J., Guo C.F., Pan T. (2021). First decade of interfacial iontronic sensing: From droplet sensors to artificial skins. Adv. Mater..

[2] Wang S., Xu P., Liu J., Wang H., Si J., Deng J., Xu M., Wang Z.L. (2023). Underwater triboelectric nanogenerator. Nano Energy.

[3] Zou Y., Tan P., Shi B., Ouyang H., Jiang D., Liu Z., Li H., Yu M., Wang C., Qu X. (2019). A bionic stretchable nanogenerator for underwater sensing and energy harvesting. Nat. Commun..

[4] Zhang Y., Li Y., Cheng R., Shen S., Yi J., Peng X., Ning C., Dong K., Wang Z.L. (2022). Underwater monitoring networks based on cable-structured triboelectric nanogenerators. Research.

[5] Zhao H., Xu M., Shu M., An J., Ding W., Liu X., Wang S., Zhao C., Yu H., Wang H. (2022). Underwater wireless communication via TENG-generated maxwell’s displacement current. Nat. Commun..

[6] Lin S., Xu L., Wang A.C., Wang Z.L. (2020). Quantifying electron-transfer in liquid-solid contact electrification and the formation of electric double-layer. Nat. Commun..

[7] Lin S., Chen X., Wang Z.L. (2021). Contact electrification at the liquid–solid interface. Chem. Rev..

[8] Xu C., Zi Y., Wang A.C., Zou H., Dai Y., He X., Wang P., Wang Y., Feng P., Li D. (2018). On the electron-transfer mechanism in the contact-electrification effect. Adv. Mater..

[9] Jbaily A., Yeung R.W. (2015). Piezoelectric devices for ocean energy: A brief survey. J. Ocean Eng. Mar. Energy.

[10] Shen F., Li Z., Guo H., Yang Z., Wu H., Wang M., Luo J., Xie S., Peng Y., Pu H. (2021). Recent advances towards ocean energy harvesting and self-powered applications based on triboelectric nanogenerators. Adv. Electron. Mater..

[11] Chen K., Ho D. (2024). Piezoionics: Mechanical-to-ionic transduction for sensing, biointerface, and energy harvesting. Aggregate.

[12] Lu C., Yu X., Chen Y., Chen X., Zhang X. (2023). Giant piezoionic effect of ultrathin MXene nanosheets toward highly-sensitive sleep apnea diagnosis. Chem. Eng. J..

[13] Chi J., Liu C., Che L., Li D., Fan K., Li Q., Yang W., Dong L., Wang G., Wang Z.L. (2022). Harvesting water-evaporation-induced electricity based on liquid–solid triboelectric nanogenerator. Adv. Sci..

[14] Dhiman P., Yavari F., Mi X., Gullapalli H., Shi Y., Ajayan P.M., Koratkar N. (2011). Harvesting energy from water flow over graphene. Nano Lett..

[15] Xue G., Xu Y., Ding T., Li J., Yin J., Fei W., Cao Y., Yu J., Yuan L., Gong L. (2017). Water-evaporation-induced electricity with nanostructured carbon materials. Nat. Nanotechnol..

[16] Yin J., Li X., Yu J., Zhang Z., Zhou J., Guo W. (2014). Generating electricity by moving a droplet of ionic liquid along graphene. Nat. Nanotechnol..

[17] Yin J., Zhang Z., Li X., Yu J., Zhou J., Chen Y., Guo W. (2014). Waving potential in graphene. Nat. Commun..

[18] Kim S.H., Haines C.S., Li N., Kim K.J., Mun T.J., Choi C., Di J., Oh Y.J., Oviedo J.P., Bykova J. (2017). Harvesting electrical energy from carbon nanotube yarn twist. Science.

[19] Yaroshchuk A. (2022). Evaporation-driven electrokinetic energy conversion: Critical review, parametric analysis and perspectives. Adv. Colloid Interface Sci..

[20] Jin H., Park J., Yoon S.G., Lee W.H., Cho Y.H., Han J., Yin Z., Kim Y.S. (2021). Verification of carrier concentration-dependent behavior in water-infiltration-induced electricity generation by ionovoltaic effect. Small.

[21] Kim J., Lee J., Kim S., Jung W. (2016). Highly increased flow-induced power generation on plasmonically carbonized single-walled carbon nanotube. ACS Appl. Mater. Interfaces.

[22] Hou Y., Zhang X.-Y., Liu C., Yin C., Yin Z. (2023). Starfish-like particles and nanowires interwoven architecture of CuO for water infiltration–induced electrical device. Nano Energy.

[23] Guo Z., Lin L., Ma J., Wang Y., Mei T., Wang X. (2023). Asymmetric GO-PPy based energy generator via synergistic flowing potential and ionovoltaic effect. J. Mater. Res. Technol..

[24] Yoon S.G., Park B.J., Jin H., Lee W.H., Han J., Cho Y.H., Yook H., Han J.W., Kim Y.S. (2021). Probing an interfacial ionic pairing-induced molecular dipole effect in ionovoltaic system. Small Methods.

[25] Cho Y.H., Jin H., Lee W.H., Han J., Jin M., Yu S., Li L., Yoon S.G., Kim Y.S. (2023). Investigation of carrier density modulation in water motion-induced ionovoltaic electricity generation. Nano Energy.

[26] Park J., Yang Y., Yoon S.G., Kim Y.S. (2019). Investigation on resistivity-dependent behavior of carbon-composite-based paintable ionovoltaic device. ACS Appl. Electron. Mater..

[27] Yang S., Su Y., Xu Y., Wu Q., Zhang Y., Raschke M.B., Ren M., Chen Y., Wang J., Guo W. (2018). Mechanism of electric power generation from ionic droplet motion on polymer supported graphene. J. Am. Chem. Soc..

[28] Nie J., Wang Z., Ren Z., Li S., Chen X., Lin Wang Z. (2019). Power generation from the interaction of a liquid droplet and a liquid membrane. Nat. Commun..

[29] Song Y., Xu B., Yuan Y., Xu H., Li D. (2019). Coalescence of a water drop with an air–liquid interface: Electric current generation and critical micelle concentration (CMC) sensing application. ACS Appl. Mater. Interfaces.

[30] Zhao X., Lu X., Zheng Q., Fang L., Zheng L., Chen X., Wang Z.L. (2021). Studying of contact electrification and electron transfer at liquid-liquid interface. Nano Energy.

[31] Lu Y., Jiang L., Yu Y., Wang D., Sun W., Liu Y., Yu J., Zhang J., Wang K., Hu H. (2022). Liquid-liquid triboelectric nanogenerator based on the immiscible interface of an aqueous two-phase system. Nat. Commun..

[32] Healy T.W., Fuerstenau D.W. (2007). The isoelectric point/point-of zero-charge of interfaces formed by aqueous solutions and nonpolar solids, liquids, and gases. J. Colloid Interface Sci..

[33] Leunissen M.E., Zwanikken J., Van Roij R., Chaikin P.M., Van Blaaderen A. (2007). Ion partitioning at the oil–water interface as a source of tunable electrostatic effects in emulsions with colloids. Phys. Chem. Chem. Phys..

[34] Silvester D.S., Jamil R., Doblinger S., Zhang Y., Atkin R., Li H. (2021). Electrical double layer structure in ionic liquids and its importance for supercapacitor, battery, sensing, and lubrication applications. J. Phys. Chem. C.

[35] Wang P., Zhang S., Zhang L., Wang L., Xue H., Wang Z.L. (2020). Non-contact and liquid–liquid interfacing triboelectric nanogenerator for self-powered water/liquid level sensing. Nano Energy.

[36] Barni F., Lewis S.W., Berti A., Miskelly G.M., Lago G. (2007). Forensic application of the luminol reaction as a presumptive test for latent blood detection. Talanta.

[37] Wang Y., Zhang S., Bai Y., Jia K., Suo Z. (2022). Chemical sensing by interfacial voltage. Cell Rep. Phys. Sci..

